# Incidence and Outcome of Acute Kidney Injury in Patients Hospitalized With Coronavirus Disease-19 at a Tertiary Care Medical Center in Saudi Arabia

**DOI:** 10.7759/cureus.18927

**Published:** 2021-10-20

**Authors:** Mahfooz A Farooqui, Alwaleed Almegren, Sattam R Binrushud, Faisal A Alnuwaiser, Nasser M Almegren, Nawaf A Alhamied, Eissa A Aloraifi, Abdullah M Alothman, Moath A Aldafas, Husam I Ardah, Fayez F Alhejaili

**Affiliations:** 1 Division of Nephrology, Department of Medicine, King Saud Bin Abdulaziz University for Health Sciences, King Abdulaziz Medical City Riyadh, Riyadh, SAU; 2 Department of Nephrology, King Abdulaziz Medical City Riyadh, Riyadh, SAU; 3 Department of Medicine, King Abdullah International Medical Research Center, Riyadh, SAU; 4 Department of Nephrology, King Saud Bin Abdulaziz University for Health Sciences College of Medicine, Riyadh, SAU; 5 Department of Nephrology, King Saud Bin Abdulaziz University for Health Sciences College of Pharmacy, Riyadh, SAU; 6 Department of Biostatistics and Epidemiology, King Abdullah International Medical Research Center, Riyadh, SAU; 7 Department of Statistics, King Saud Bin Abdulaziz University for Health Sciences, Riyadh, SAU; 8 Department of Biostatistics, King Abdulaziz Medical City Riyadh, Riyadh, SAU

**Keywords:** 30 day mortality, hospitalized, sars-cov-2, covid-19, acute kidney injury

## Abstract

Introduction

The systemic acute respiratory syndrome coronavirus (SARS-CoV-2) has been associated with acute kidney injury (AKI). We retrospectively studied the incidence and outcome of AKI in patients hospitalized with COVID-19 at King Abdulaziz Medical City (KAMC) Riyadh, Kingdom of Saudi Arabia.

Methods

A retrospective cohort study was conducted after ethical approval from the institutional review board of King Abdullah International Medical Research Center (KAIMRC). Subjects were identified by Data Management Office of KAIMRC. The data were extracted from electronic medical records using a customized data collection sheet.

The study included all adult patients (>18 years) who tested positive for COVID-19 by polymerase chain reaction and were admitted at KAMC from March 2020 until the end of September 2020. Patients with a history of end-stage kidney diseases and patients where adequate data were not available to establish diagnosis of AKI were excluded.

Patient demographics, comorbid conditions, medications, use of mechanical ventilation, and 30-day mortality were recorded.

Results

During the study period (01 March 2020 to 30 September 2020) 1293 patients were hospitalized at KAMC with the diagnosis of COVID-19. After excluding the patients who met the exclusion criteria, data were collected for 1025 patients [male 582 (56.8%); female 443 (43.2%)]. On univariate analysis, increasing age, male gender, use of angiotensin-converting enzyme inhibitors, angiotensin receptor blockers, diuretics, and vasopressors, presence of chronic kidney disease, coronary artery disease, chronic obstructive pulmonary disease, dyslipidemia, diabetes mellitus, heart failure, and hypertension, kidney transplant status, and mechanical ventilation were associated with development of AKI.

On multivariate logistic regression analysis, independent predictors of AKI were restricted to increasing age, presence of chronic kidney disease, hypertension, kidney transplant status, use of vasopressors, and mechanical ventilation.

For patients who developed AKI, 30-day mortality was 40.7% compared to 3.7% for those who did not develop AKI (p<0.001).

Conclusion

For hospitalized patients with COVID-19, we observed an incidence of AKI of 36%. Increasing age, presence of chronic kidney disease and hypertension, kidney transplant status, use of vasopressors, and mechanical ventilation were independently associated with development of AKI. Presence of AKI was associated with higher 30-day mortality (40.7% vs 3.7%).

## Introduction

The severe acute respiratory syndrome coronavirus 2 (SARS-CoV-2) was first reported in Wuhan, China in late 2019. The virus rapidly spread to cause one of the largest pandemics in over a century. The virus has caused global travel disruptions and large-scale economic losses, in addition to huge loss of lives and overwhelmed healthcare services in developing as well as developed countries. The virus predominantly involved the respiratory system but reports of proteinuria, hematuria, and acute kidney injury (AKI) by the virus began to emerge early on. A wide range of pathophysiological mechanisms of renal involvement in COVID-19 have been described.

In the Kingdom of Saudi Arabia, the first case of COVID-19 was reported on 02 March 2020. As the number and severity of patients affected with the disease increased, an increase in cases with AKI was also observed.

## Materials and methods

We retrospectively studied the incidence, risk factors, and outcomes associated with AKI among hospitalized patients with COVID-19 at King Abdulaziz Medical City (KAMC) in Riyadh, Kingdom of Saudi Arabia. KAMC is a major tertiary care medical center with more than 1600 general inpatient beds and more than 120 critical care beds. The study was approved by the Institutional Review Board Committee (IRBC) of King Abdullah International Medical Research Center on 25 November 2020 (Study number RC20/593/R). Data management department identified all patients admitted to the KAMC Riyadh with a diagnosis of COVID-19, between 02 March 2020 (when first case was diagnosed in the Kingdom of Saudi Arabia) to end of September 2020. All cases were diagnosed based on COVID-19 polymerase chain reaction. Patients less than 18 years old, patients with end-stage kidney diseases, and patients with no serum creatinine or only one serum creatinine level available were excluded. The data were extracted from electronic medical records using a customized data collection sheet. The collected variables included patient’s demographics (gender, age, height, weight, and BMI), comorbid conditions such as hypertension (HTN), diabetes mellitus (DM), asthma, coronary artery disease (CAD), heart failure (HF), dyslipidemia (DLP), chronic obstructive pulmonary disease (COPD), and chronic kidney disease (CKD), need for mechanical ventilation, and 30-day mortality.

Means and proportions of the study participants were calculated to characterize the study participants, overall and in groups. The primary outcome variable was development of AKI. To determine the factors associated with developing AKI, the study participants were divided into two groups based on whether a patient developed AKI. The two groups (AKI, No AKI) were compared using chi-square or Fisher's exact test for categorical factors and t-test or Kruskal Wallis test for continuous variables as appropriate. Then, multivariate logistic regression analysis was used to identify independent risk factors. In the multivariate logistic regression model, the probability of developing AKI was modeled as the dependent variable, and all variables that were found to be associated with developing AKI as well as the potential confounders such as gender, age, and DM as the independent variables. For survival analyses, we generated Kaplan-Meier survival curves, and comparison was done using the log-rank test. Another logistic regression model adjusted for covariates (adjusted for age, sex, and comorbidities) was used to estimate the adjusted odds ratio for death in patients with AKI versus without AKI. Covariates were chosen based on univariate testing and physician input. Level of significance was declared at α =0.05. Statistical analysis was conducted using SAS 9.4 (SAS Institute Inc., Cary, NC, USA).

## Results

During the study period (01 March 2020 to 30 September 2020) 1293 patients were hospitalized at KAMC with the diagnosis of COVID-19. After excluding the patients who met the exclusion criteria, data were collected for 1025 patients [male 582 (56.8%); female 443 (43.2%)]. On univariate analysis, age (p<0.0001), male gender (p<0.0001), use of angiotensin-converting enzyme inhibitors (p<0.0038), angiotensin receptor blockers (p<0.0001), diuretics (p<0.0001), and vasopressors (p<0.0001), presence of CKD (p<0.0001), CAD (p<0.0001), COPD (p=0.0008), DLP (p=0.0008), DM (p<0.0001), HF (p<0.0001), and HTN (<0.0001), kidney transplant status (p=0.0112), and mechanical ventilation (p,0.0001) were associated with development of AKI (Table 1).

**Table 1:** Association of participants' characteristics with presence of AKI Denominator of the percentage is the total number of subjects in each group. *t-Test/^Wilcoxon rank sum test is used to calculate the p-value. **Chi-square test is used to calculate the p-value. ^^Fisher's exact test is used to calculate the p-value. AKI, acute kidney injury.

Name of former variable		AKI=369	No AKI=656	Total=1025	p-Value
Gender	Female n (%)	112 (30.4)	331 (50.5)	443 (43.2)	<0.0001
	Male n (%)	257 (69.6)	325 (49.5)	582 (56.8)	<0.0001
Age at diagnosis of COVID	Mean (SD)	64.6 (15.36)	50.8 (18.29)	55.8 (18.52)	<0.0001
Body mass index	Mean (SD)	29.7 (7.19)	30.0 (6.51)	29.9 (6.76)	0.1870^
Diabetes mellitus	n (%)	259 (70.2)	272 (41.5)	531 (51.8)	<0.0001
Hypertension	n (%)	269 (73.9)	263 (40.2)	532 (52.2)	<0.0001
Coronary artery disease	n (%)	79 (21.4)	76 (11.6)	155 (15.1)	<0.0001
Dyslipidemia	n (%)	149 (40.4)	197 (30.0)	346 (33.8)	0.0008**
Heart failure	n (%)	62 (16.8)	44 (6.7)	106 (10.4)	<0.0001
Asthma	n (%)	41 (11.1)	56 (8.5)	97 (9.5)	0.1765**
Chronic obstructive pulmonary disease	n (%)	20 (5.4)	11 (1.7)	31 (3.0)	0.0008**
Chronic kidney disease	n (%)	168 (45.5)	133 (20.3)	301 (29.4)	<0.0001
Kidney transplant	n (%)	9 (2.4)	3 (0.5)	12 (1.2)	0.0112^^
Angiotensin-converting enzyme inhibitors	n (%)	80 (21.7)	96 (14.6)	176 (17.2)	0.0038**
Angiotensin receptor blockers	n (%)	84 (22.8)	74 (11.3)	158 (15.4)	<0.0001
Diuretics	n (%)	272 (73.7)	198 (30.2)	470 (45.9)	<0.0001
Vasopressor	n (%)	207 (56.1)	42 (6.4)	249 (24.3)	<0.0001
Mechanical ventilation	n (%)	208 (56.4)	73 (11.1)	281 (27.4)	<0.0001
30-Day COVID mortality	n (%)	150 (40.7)	24 (3.7)	174 (17.0)	<0.0001

On multivariate logistic regression analysis, independent predictors of AKI were restricted to increasing age (p=0.0041), presence of CKD (p=0.0169) and HTN (p=0.0021), kidney transplant status (p=0.0216), use of vasopressors (p<0.0001), and mechanical ventilation (p=0.0252) (Table 2).

**Table 2:** Independent predictors associated with presence of AKI DM, diabetes mellitus; HTN, hypertension; CAD, coronary artery disease; DL, dyslipidemia; HF, heart failure; COPD, chronic obstructive pulmonary disease; CKD, chronic kidney disease; ACE, angiotensin-converting enzyme inhibitors; ARB, angiotensin receptor blockers.

Effect	Beta	Standard error	Odds ratio	95% Confidence interval	p-Value
Female vs male	-0.2996	0.2030	0.741	(0.50, 1.10)	0.1400
Age at diagnosis of COVID-19	0.0176	0.00686	1.018	(1.00, 1.03)	0.0104
DM vs No DM	0.1214	0.2400	1.129	(0.71, 1.81)	0.6130
HTN vs No HTN	0.8213	0.2676	2.273	(1.35, 3.84)	0.0021
CAD vs No CAD	-0.2015	0.2701	0.818	(0.48, 1.39)	0.4556
DLP vs No DLP	-0.3951	0.2263	0.674	(0.43, 1.05)	0.0808
HF vs No HF	0.2116	0.3171	1.236	(0.66, 2.30)	0.5046
COPD vs No COPD	0.3240	0.5527	1.383	(0.47, 4.09)	0.5578
CKD vs No CKD	0.5567	0.2330	1.745	(1.11, 2.75)	0.0169
Kidney transplant vs No kidney transplant	1.7765	0.7730	5.909	(1.30, 26.89)	0.0216
ACE vs No ACE	0.0704	0.2570	1.073	0.65, 1.78)	0.7841
ARB vs No ARB	0.3966	0.2716	1.487	(0.87, 2.53)	0.1443
Diuretics vs No diuretics	0.3639	0.2106	1.439	(0.95, 2.17)	0.0840
Vasopressor vs No vasopressor	2.3506	0.3019	10.491	(5.81, 18.96)	<0.0001
Mechanical ventilation vs No mechanical ventilation	0.6480	0.2895	0.523	(0.30, 0.92)	0.0252

**Figure 1:**
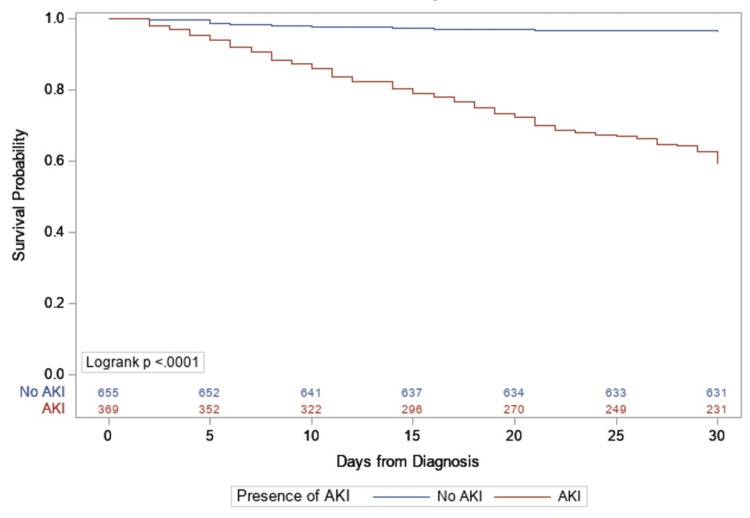
Kaplan-Meier cumulative incidence curve of mortality by presence of AKI with number of subjects at risk. AKI, acute kidney injury

Presence of AKI was associated with higher 30-day mortality. For patients who developed AKI, 30-day mortality was 40.7% compared to 3.7% for those who did not develop AKI (p<0.001). Figure 1 shows Kaplan-Meier curve of cumulative mortality by presence of AKI.

**Figure 2:**
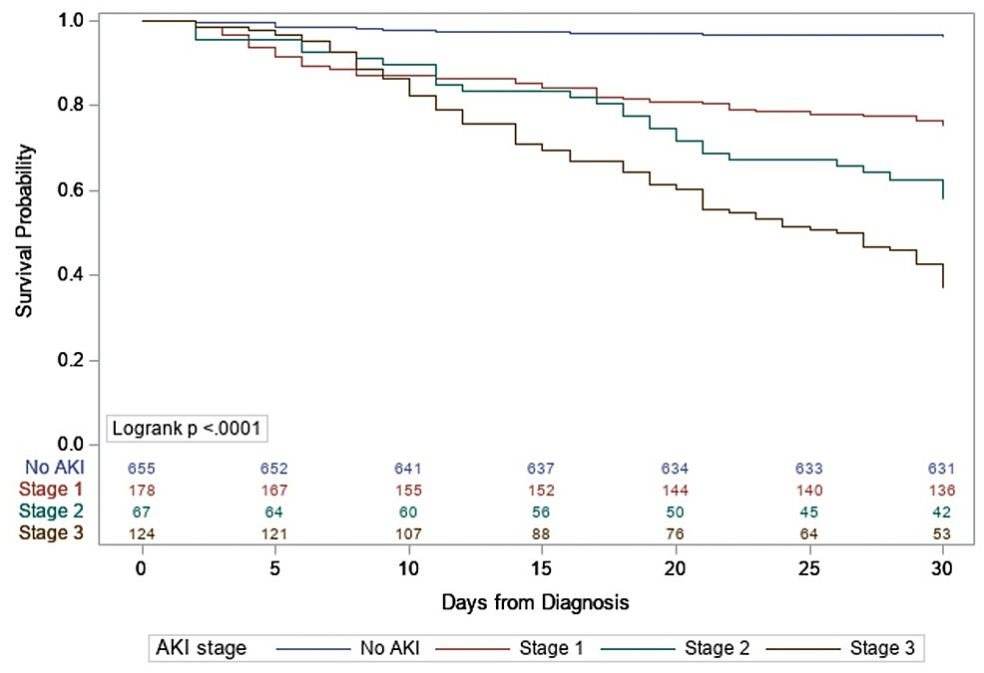
Kaplan-Meier cumulative incidence curve of mortality by stage of AKI with number of subjects at risk. AKI, acute kidney injury

We used Kidney Disease Improving Global Outcomes (KDIGO) Acute Kidney Injury criteria for staging the severity of AKI. We observed that progressively severe AKI stages were associated with higher 30-day mortality. Figure 2 shows Kaplan-Meier cumulative incidence curve of mortality by stages of AKI.

## Discussion

COVID-19 pandemic has wreaked havoc with significant morbidity and mortality. The disease, since it was first reported from Wuhan, China, has spread to all over the world. It has overwhelmed healthcare services and disrupted travel. Businesses were shut down bringing economies of many countries to their knees. Initial reports from Wuhan suggested that AKI is not associated with COVID-19 [1]. As the disease case load increased, reports of AKI started to emerge [2] early during the pandemic. Clinical features [3-7] and pathophysiology of kidney involvement due to COVID-19 were studied and reported [7,8]. Reports of proteinuria, hematuria, and kidney failure emerged. Post-mortem examination [9] and kidney biopsy results [10,11] were reported and various mechanisms for kidney involvement were proposed [12-14]. 

AKI formed bulk of the involvement and was described from various centers.

In the Kingdom of Saudi Arabia, the first case of COVID-19 was reported on 02 March 2020. The number of cases increased and resulted in efforts to contain the disease by mandatory use of mask and social distancing along with restricting movement of people and large gatherings. Healthcare services expanded to reveal their adaptability by converting general wards to high-dependency and critical care units. A high incidence of AKI was soon recognized in patients affected with COVID-19 as number of patients requiring continuous renal replacement therapy (RRT) and bed-side acute hemodialysis surged. Like many other countries, renal unit staff struggled to keep with this tsunami of AKI especially as many of their colleagues were also affected and some were not able to return to work due to travel restrictions [15].

Reported incidence of AKI has varied widely from different centers. It has varied from as low as 4.5% to as high as 69% [16-19]. It is expected that during an active wave of the virus the number of patients in the given community would be higher and hence the number of AKI will also be higher. It is also obvious that a study limited to critical care units will have higher incidence of AKI when compared to general inpatients. Incidence of AKI will be even lower if all COVID-19-positive patients are included as many cases remain asymptomatic or minimally symptomatic. Identifying patient group with high risk of development of AKI, early recognition of the AKI, and employing prompt preventive or therapeutic maneuvers may potentially help in minimizing morbidity and mortality. We selected a longer period of study, which included peak of the number of hospitalized cases as well as a period when the COVID-19 was relatively controlled. We included all inpatients to include critical and non-critical patients. In our experience, above 70-year age group was affected the most despite the fact that, unlike many western countries, this represents the smallest group in Saudi Arabia [20]. 

In patients with COVID-19, multiple etiologies are at play to produce AKI. Two distinctive phenotypes of AKI, AKI-early and AKI-late, have been suggested. These two types are reported to have difference in outcome [2]. In early stages, fever and diarrhea often result in dehydration. Use of non-steroidal anti-inflammatory drugs for symptomatic control of fever may predispose these individuals to AKI. Additionally, It is often difficult to distinguish infection or presence of pulmonary edema on chest x-ray [21]. Many a times diuretics are chosen to improve oxygenation in hypoxic patients to eliminate any contribution of HF. It is not customary to monitor central venous pressures in non-critical-care settings. Quickly obtaining urine studies before administration of diuretics and utilization of non-invasive techniques, such as point-of-care ultrasound, may help in guiding fluid therapy in this group of patients [22,23]. Readily reversible causes, such as urinary obstruction, may also be picked up on bed-side ultrasound. Efforts to avoid hypotension by tapering or holding anti-hypertensive medications before significant hypotension develops may avoid ischemic kidney injury. Disordered coagulation with micro-thrombosis and embolism is well described in patients with COVID-19. However, the role of anticoagulation in preventing AKI is unclear. Supportive RRT for patients with established AKI is of paramount importance. Patients who develop AKI and particularly those requiring RRT are reported to have worse outcome [24,25]. All forms of RRTs have been employed. Peritoneal dialysis has been tried successfully and remains a valuable tool especially during periods of acute surge in the number of patients requiring RRT [26,27]. The modality of RRT is often selected based on patient characteristics and resource availability. Prolonged intermittent renal replacement therapy (PIRRT) may be employed for critically ill patients in resource-limited settings. Critically ill patients with COVID-19 may go through phases of improvement and deterioration due to secondary infections. Their dialysis modality is often switched between CRRT to PIRRT to conventional hemodialysis. A review of medications is required each time the modality is switched as the clearance of medications may vary with the modality. Similarly, the nutritional requirements and electrolyte replacements may change in patients with sepsis with varying modalities of RRT. Signs of renal recovery should be monitored closely and RRT should be stopped when renal recovery is noted. Optimum care of these patients requires highly skilled and coordinated care delivery by the nurse, intensivist, nephrologist, infectious disease clinician, clinical pharmacologist, and dietitian.

## Conclusions

AKI is a common complication of COVID-19 in hospitalized patients and is associated with significant morbidity and mortality. We observed that in hospitalized patients with COVID-19, the incidence of AKI was 36%. Older age, presence of CKD and HTN, kidney transplant status, use of vasopressors, and mechanical ventilation were independently associated with development of AKI. Presence of AKI was associated with higher 30-day mortality (40.7% vs 3.7%). Furthermore, severity of AKI, as determined by KDIGO stage, was also associated with higher mortality.

Prospective studies may reveal how vaccination and newer therapeutic agents against COVID-19 will change the incidence of AKI and improve mortality.
